# Acetolactate Synthase-Inhibiting Gametocide Amidosulfuron Causes Chloroplast Destruction, Tissue Autophagy, and Elevation of Ethylene Release in Rapeseed

**DOI:** 10.3389/fpls.2017.01625

**Published:** 2017-09-21

**Authors:** Xi-Qiong Liu, Cheng-Yu Yu, Jun-Gang Dong, Sheng-Wu Hu, Ai-Xia Xu

**Affiliations:** Department of Plant Science and Technology, College of Agronomy, Northwest A&F University Yangling, China

**Keywords:** *Brassica napus*, gametocide, acetolactate synthase, amidosulfuron, plastid, male sterility

## Abstract

**Background:** Acetolactate synthase (ALS)-inhibiting herbicides amidosulfuron (Hoestar) is an efficient gametocide that can induce male sterility in rapeseed (*Brassica napus* L.). We conducted an integrated study of cytological, transcriptomic, and physiological analysis to decipher the gametocidal effect of amidosulfuron.

**Results:** In the first several days after exposure to amidosulfuron at a gametocidal dose of ca. 1 μg per plant, the plants showed the earliest symptoms including short retard of raceme elongation, slight chlorosis on leaf, and decrease of photosynthesis rate. Chloroplasts in leaf and anther epidermis, and tapetal plastids were deformed. Both tapetal cell and uni-nucleate microspore showed autophagic vacuoles and degenerated quickly. The amidosulfuron treatment caused reduction of photosynthetic rate and the contents of leaf chlorophyll, soluble sugar and pyruvate, as well as content alteration of several free amino acids in the treated plants. A comparison of transcriptomic profiling data of the young flower buds of the treated plants with the control identified 142 up-regulated and 201 down-regulated differential expression transcripts with functional annotations. Down-regulation of several interesting genes encoding PAIR1, SDS, PPD2, HFM1, CSTF77, A6, ALA6, UGE1, FLA20, A9, bHLH91, and putative cell wall protein LOC106368794, and up-regulation of *autophagy-related protein ATG8A* indicated functional abnormalities about cell cycle, cell wall formation, chloroplast structure, and tissue autophagy. Ethylene-responsive transcription factor RAP2-11-like was up-regulated in the flower buds and ethylene release rate was also elevated. The transcriptional regulation in the amidosulfuron-treated plants was in line with the cytological and physiological changes.

**Conclusions:** The results suggested that metabolic decrease related to photosynthesis and energy supply are associated with male sterility induced by amidosulfuron. The results provide insights into the molecular mechanisms of gametocide-induced male sterility and expand the knowledge on the transcriptomic complexity of the plants exposure to sulfonylurea herbicide.

## Introduction

Significant heterosis (hybrid vigor) for seed yield and other traits in rapeseed (*Brassica napus* L.), which is one of the important sources of edible oil in the world after soybean and palm, is well-documented (Yu et al., 2005). A key step in the utilization of heterosis is to control out-crossing in the maternal line, which can be achieved by either chemical induced or inheritable male sterility. Although, some inheritable male sterility systems including genic male sterility and cytoplasmic male sterility are often used, they require time- and labor-consuming pre-breeding efforts to create different lines that are named male sterile line, maintainer, and restorer. Some male sterility-induced systems also showed penalties on seed-set, quality traits, and/or disease resistance (Chakrabarty et al., 2015; Xia et al., 2016; Dey et al., 2017). The chemically induced male sterility (CIMS) approach employs male gametocide to induce male sterility in sensitive plant and therefore selfing of the plant is avoided and outcrossing promoted. This method does not require too much pre-breeding work. Nevertheless, obtention of highly efficient gametocides is the first task in a breeding scheme using CIMS. Some herbicides that inhibit acetolactate synthase (ALS; EC 4.1.3.18), such as tribenuron-methyl (Express) and amidosulfuron (Hoestar/Gratil), were showed to be able to induce complete male sterility in many cruciferous species when they were applied at trace amounts (about 1% dosage recommended for weed control; Yu et al., 2006, 2009, 2017; Yu and He, 2014; Li et al., 2015). In the last decade, over 20 commercial hybrid rapeseed varieties based on CIMS have been registered in China, for examples, Shaanyou1209, Zhongyouza19, Qinyou33, and Yuhuang1. Owing to the wide application of acetolactate synthase-inhibiting gametocides in China, CIMS is becoming an important approach for fast utilization of the newly derived breeding lines.

ALS is the key enzyme that catalyzes the reaction to synthesize three branched-chain amino acids (BCAAs) including leucine, valine, and isoleucine in plants and some microbes. ALS-inhibitors belonging to sulfonylurea, imidazolinone, triazolopyrimidine, and other chemical families are widely used for weed control because of their large spectrum of control activity, high crop selectivity, and low mammalian toxicity (Zhou et al., 2007). Oilseed rape is very susceptible to sulfonylurea herbicides and thus most sulfonylurea herbicides have strong gametocidal effect on it (Yu and He, 2014). Amidosulfuron and tribenuron- methyl are of the most useful gametocides for *B. napus* among sulfonylurea herbicides. Our findings of gametocidal effect of those chemicals provided a novel function of ALS-inhibiting herbicides.

Although, ALS had been proven to be the site of action (McCourt et al., 2006), the mechanism through ALS-inhibitors cause phytotoxicity has progressed unprecedentedly in the last two decades. Physiology, genetics, molecular, and chemical structure aspects caused by ALS-inhibiting herbicides have been discussed (Reviewed by Zhou et al., 2007). Except BCAAs starvation (Ray, 1984), other hypothesis about secondary effects of ALS inhibition, such as accumulation of pyruvate and 2-ketobutyrate or 2-aminobutyrate, inhibition of DNA synthesis, disruption of photoassimilate translocation, and anaerobic respiration, have also been implicated in the mechanism of plant death caused by ALS-inhibiting herbicides (reviewed by Zhou et al., 2007). However, there are still some disputations and doubts on the precise mechanisms that need further probing into (Zhou et al., 2007).

A few of studies involving transcriptional analyses had been undertaken to decipher the diagnostic detection, mode of action, or detoxification mechanisms of ALS-inhibitors (Glombitza et al., 2004; Pasquer et al., 2006; Manabe et al., 2007; Das et al., 2010). Time course analysis of imidazolinone-sensitive and resistant mutants in *Arabidopsis thaliana* identified early-response genes related to detoxification while later-response genes participated in biosynthesis of amino acids, secondary metabolites and tRNAs (Manabe et al., 2007). A focused DNA array comprising of 267 genes related to secondary metabolism could be used to differentiate the effects of primisulfuron and prosulfuron exposure on *A. thaliana* (Glombitza et al., 2004). Many effects of the ALS-inhibiting herbicides had been revealed based on transcriptional changes and a few genes were found to be able to differentiate responses to closely related herbicides in *A. thaliana* and *B. napus* (Das et al., 2010). Except the above-mentioned reports of gene expression analysis of plants to different ALS-inhibiting herbicides (Das et al., 2010), few studies (Cheng et al., 2013; Li et al., 2015) were carried out to understand the mechanism of inducing male sterility by trace amounts of ALS-inhibiting gametocide. Thus, the inducing mechanism of male sterility in the susceptible plants by these herbicides remains unknown. For gametocidal effect of sub-herbicidal level of tribenuron-methyl, monosulfuron ester sodium (MES, belongs to sulfonylurea family) and imazethapyr (belongs to imidazolinone family), blocks of carbohydrate and lipid metabolism and autophagic cell death were suggested (Qian et al., 2011; Li et al., 2015; Zhao et al., 2015). Transcriptome analysis using microarray (Li et al., 2015) identified 1,501 differentially expressed transcripts (DETs) in the leave and anthers of the treated plants. Trace concentrations of herbicide imazethapyr, which can be used as a gametocide for both rapeseed and wheat (Yu, 2009), inhibited some key genes regulating anther and pollen biosynthesis in *A. thaliana* (Qian et al., 2015).

Sulfonylurea herbicides such as amidosulfuron are fully systemic and slower to act than traditional herbicides, moving throughout the weed and resulting in high levels of control. Amidosulfuron had similar gametocidal effect among different cultivars in *B. napus*, although the influences of plant biomass and leaf cuticle were inevitable (Yu et al., 2009; Yu and He, 2014). Foliar application of amidosulfuron at the doses from 60 to 90 mg/ha resulted in over 95% male sterility in the treated *B. napus* plants as well as lower phytotoxicity on pistil fertility (Yu et al., 2009; Yu and He, 2014). The objective of this study was to uncover the physiological and transcriptional responses of rapeseed to amidosulfuron exposure and to find possible associations of them with amidosulfuron phytotoxic effects, particularly male sterility. We investigated the morphological, physiological, and cytological differences between the male sterile amidosulfuron-treated plants and fertile control at different developmental stages. We also conducted a transcriptomic comparison between juvenile flower buds of the treated plants and the control. The cytological and transcriptomic alterations in the treated plants were in line with reduction of chlorophyll, soluble sugar, pyruvate and photosynthetic rate, and increase of ethylene release rate. This results gave us some clues to decipher the gametocidal mechanism of amidosulfuron and other ALS-inhibitors.

## Materials and methods

### Plant material and amidosulfuron treatment

The plants of winter rapeseed cv. Qinyou3 were grown in the experimental field of Northwest A&F University from mid-September to late May of the next year. In the spring, the bolting plants with the longest flower bud ≤3 mm were foliar-sprayed with 0.2 mg a.i./L amidosulfuron (Gratil, 75% water dispersible granule) solution for about 5 mL per plant. The solution was added with a surfactant sodium alkylethersulfate (Biopower™, Bayer AG, Monheim am Rhein, Germany) at a concentration of 0.2 mL/L. This treatment had at least three replicates, each containing of about 70 plants. An additional set of plants, used as the negative control, were sprayed only with the water containing the surfactant.

### *In vivo* activity of ALS

In order to know how the gametocide amidosulfuron affect plant ALS proteins *in vivo*, the activities of ALS enzymes in mature leaf and young flower bud (length ≤ 3 mm) were assayed0, 1, 4, 7, and 13 days after treatment (DAT). The *in vivo* assay ALS activity, which was represented by accumulation of the substrate acetolactate, can monitor the activity inhibition by the ALS-inhibiting herbicide that is absorbed and even partially degraded by plant (Gerwick et al., 1993; Simpson et al., 1995). It had advantages over *in vitro* assay because the latter method is used to compare the activity of ALS proteins, isolated from different genotypes, reacting with exogenous substrate, and is not appropriate in cases of metabolic detoxification of the herbicide, unsteady supply of substrate, or altered expression of the *ALS* genes in the treated plants. In brief, the plants in a treatment were sprayed with 0.5 mol/L of 1,1-cyclopropanedicarboxylate (CPCA), a specific inhibitor of ketol-acid reductoisomerase (KARI) subsequent to ALS, to accumulate ALS enzyme substrate acetolactate. After 24 h, leaf or flower bud samples were collected from the CPCA treated plants and 1 g sample was extracted with 5 mL of 0.05 M sodium phosphate buffer (pH 7.2) and centrifuged 10 min at 8,000 × *g*. 0.5 mL supernatant was incubated at 60°C for 10 min. 0.1 mL of 1 M sulfuric acid was added to stop the catalysis of ALS enzyme and the incubation was maintained for 30 min to generate acetoin. Then 1 mL of 0.5% (W/V) creatine (prepared in 2 M NaOH) and 1 mL of 5% (W/V) alpha naphthol (in 2 M NaOH) were added and kept at 37°C for 30 min to transform acetoin into a red compound. Supernatant was obtained after an instant centrifugation and detected by using a spectrophotometer. Absorbance of supernatant was measured at 530 nm (A530) and then ALS activity was calculated as A530 per hour by 1 g fresh sample. Each samples had three replicates and Student's *t*-test was used for comparisons of the same tissues collected on the same day.

### Physiological traits

The photosynthetic rate of the plants 0, 2, 4, 6 DAT was measured using a Li-6400 portable photosynthetic system (Li-COR Inc., Linco, NE, USA). The upper mature leave of five plants in each treatment were used for the measurement. The measurements were done at 28°C with supply of 500 μmol/(m^2^·s) CO_2_ and 1,000 μmol/(m^2^·s) artificial light. Content of soluble sugar, chlorophyll, and pyruvate were determined in the upper leaf of the plants 1, 3, and 5 DAT using colorimetric methods described by Chen (2002). Student's *t*-test was used for pairwise comparisons of these variables.

### Assay of ethylene release rate

0.5 g sample of small flower buds (length ≤3.0 mm) with three replicates were collected 1, 3, and 5 DAT. The samples were put in 25 mL bottles with rubber stoppers and then the bottles were sealed by water. The gas released by each sample, obtained after a preservation of 12 h in dark, was assayed by a GC2010Plus gas chromatographer (Shimadzu, Tokyo, Japan). One milliliter of collected gas was injected into the chromatographer at a speed of 1.5 mL/min. The carrier gas N_2_ and the make-up gas H_2_ were at a same flow rate of 35 mL/min. The temperature of injector, detector, and oven were 100, 150, and 70°C, respectively. A hydrogen ion flame detector and stainless steel column packed with GDX-502 were used in the chromatographer. The retention time was 2.5 min.

### Content of free amino acid

The stamen of 2–3 mm long flower buds of the plants 3 DAT were sampled on ice. A 1.0 g sample was homogenized in 80% ethanol. The homogenates were filtered, transferred into an evaporating dish, and dried on water bath at 80°C. The amino acid sediment was dissolved in a mix of 15 mL of sodium citrate buffer (pH 2.2) and 5 mL of 5% sulfosalicylic acid, and centrifuged 15 min at 10,000 × *g*. Free amino acid composition in the supernatant was detected at a Beckman 121 MB amino acid analyzer (Beckman Instruments Inc., CA, USA). Both the amidosulfuron treatment and the control had three independent biological replicates.

### Cytological study

When the treated plants were blossoming, aceto-carmine staining of anther was performed to examine the microspore and pollen developmental stage under a light microscopy. Flower buds from male fertile and sterile plants were collected according to their developmental stages. The leave and anthers from these grouped buds were fixed by glutaraldehyde-osmium tetroxide, embedded in epoxy resin. The specimens were cut into 0.05 μm ultrathin sections, stained in uranyl acetate -lead citrate solution, and then observed under a transmission electron microscopy (TEM). The detailed process of section preparation was described in a previous study (Yu et al., 2016). The structural abnormality that was observed in the most samples of the same developmental stage in the treated plants were recorded.

### RNA extraction

Based on cytological observation, we knew that the key stage sensitive to gametocide is pollen mother cell (PMC) to uni-nucleate microspore. Thus, the corresponding young flower buds (length ≤ 3 mm) from the plants 5 DAT and the control plants (designed as DAT1, DAT2, DAT3 for the treatment and F1, F2, F3 for the control) were dissected, put into RNAlater (LC Science, Houston, USA) for 24 h, and stored at −80°C. Total RNAs were extracted using the Trizol® Reagent (Invitrogen, USA) and purified using TRK1001 Kit (LC Science, Houston, USA). The purity and integrity of total RNA samples were analyzed by Bioanalyzer 2100 with RNA 6000 Nano LabChip Kit (Agilent, CA, USA). We used 10 μg total RNA from each sample to create normalized cDNAs. Both the amidosulfuron treatment and the control had three independent biological replicates.

### RNA sequencing

Digital gene-expression tag profiling (DGE) is a simple and cost-efficient method to analyse transcriptome using Illumina Solexa technology. Two groups of DGE libraries, each in three biological replicates (DAT1, DAT2, DAT3 and F1, F2, F3), were constructed using the above-mentioned cDNAs of young flower buds with Illumina's Digital Gene Expression Tag Profiling Kit according to the manufacturer's protocol. We performed the single end sequencing on an Illumina GAIIX platform following the vendor's protocol. The adaptor sequences, tags with low quality sequences, and unknown nucleotides N were filtered from the raw data to obtain clean reads with 36 nt in length. The clean reads were deposited in NCBI under Gene Expression Omnibus accession GSE69679. All the reads were aligned with *Brassica* genome in both JGI database (http://genome.jgi.doe.gov/) and NCBI database (http://www.ncbi.nlm.nih.gov/) by using Bowtie2 v2.1.0 (http://sourceforge.net/projects/bowtie-bio/files/bowtie2/2.1.0/), and only 1 bp mismatch was allowed. We also aligned the clean tags with a previous transcriptome assembly of rapeseed flower buds. The assembly contained 135,702 unigenes (file Trinity_rapeseed.fsa under TSA accession GDFQ00000000), most of which had been successfully annotated in various databases including NCBI non-redundant, JGI, Pfam, SwissProt, and Clusters of Orthologous Groups of proteins (unpublished). The number of perfect reads matching to each unigene was normalized to the number of Reads Per Kilobase of exon model per Million mapped reads (RPKM). The low-frequency transcripts with the 3rd quartile of counts in all samples < 10 were filtered. Based on expression levels, the significant DETs among the two groups of samples were identified with criteria of false discovery rate (FDR) ≤0.05 and fold-change ≥2. The sequence of each DET was searched in NCBI nucleotide collection database by BLAST and the function was annotated by the references in Uniprot (http://www.uniprot.org/) and gene ontology (GO) database (http://www.geneontology.org/). The hierarchical cluster of the DETs was performed by using Cluster 3.0 (http://bonsai.hgc.jp/~mdehoon/software/cluster/software.htm). The GO networks for down-regulated and up-regulated genes were drew by the BiNGO plugin of Cytoscape software (http://www.cytoscape.org/). All possible interactions among the DETs were revealed based on the known and predicted information of Arabidopsis in STRING (https://string-db.org/).

### Gene expression analysis by quantitative real-time PCR (QRT-PCR)

Some tissues including upper leaf, small buds of ca. 1 mm length, medium buds of ca. 3 mm, large buds of ca. 5 mm, and anthers of the medium buds were collected from at least 5 plants 3 DAT, with three independent biological replicates for each sample. The RNA was extracted and cDNA was synthesized with 250 ng of total RNA using PrimeScript RT reagent Kit (Takara, Otsu, Japan). Gene-specific primers (Table S1) were designed for two function-important *ALS* loci (*ALS1* and *ALS3*; Zhao et al., 2015) and 13 selected DETs according to the unigene sequences using the Beacon Designer 7.0 (Bio-Rad, CA, USA). Real-time PCR assays in triplicate were performed using SYBR Green PCR Master Mix (Applied Biosystems, CA, USA) on a QuantStudio 3 thermal cycler (Thermo Fisher Scientific, CA, USA). Tissues from water-treated plants were employed as negative control and *B. napus* beta-actin7 gene was used as the internal control for data normalization. The relative fold change of each gene in the different samples was calculated by using threshold cycle relative quantification parameter 2^−Δ^CT (Schmittgen and Livak, 2008), where ΔCT = (Ct_gene_ - CT_actin_). Student's *t*-test was used for pairwise comparisons between the same type of tissues.

## Results

### Morphological changes of rapeseed exposed to gametocide amidosulfuron

**Figure 1:**
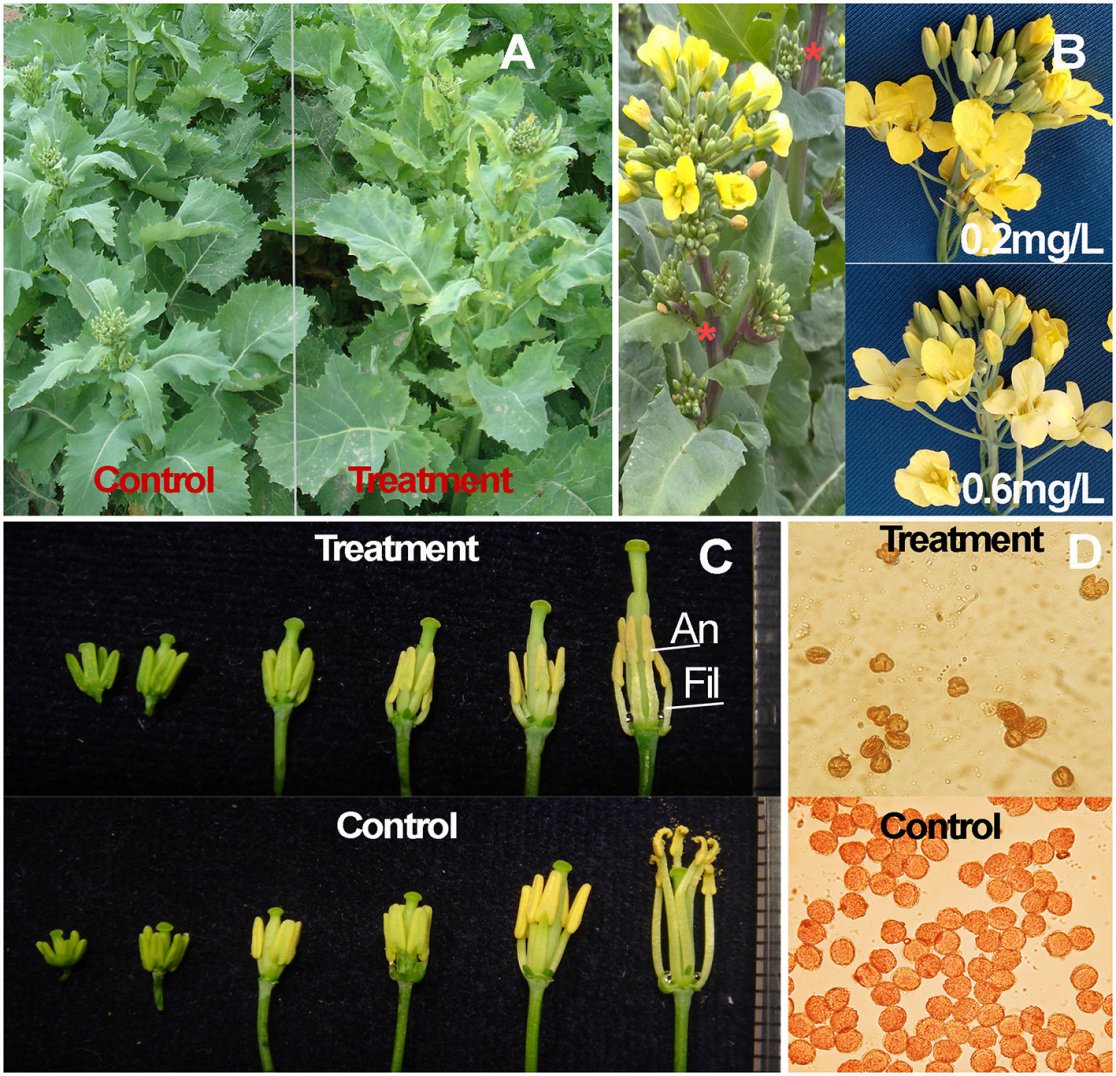
Morphological characteristics and pollen grains of amidosulfuron-treated plants. **(A)** Chlorosis on the treated plants 5 DAT. **(B)** Inflorescence with anthocyanin accumulation (asterisk) and petal depigment due to higher dose (0.6 mg/L) exposure. **(C)** Size reduction of filament and anther in the treated plant after uni-nucleate microspore stage (bud length about 3 mm). **(D)** Viable pollen grains in the control are deep stained by the aceto-carmine, while the unviable pollen grains in the treatment are not stained. An, anther; Fil, filament.

The exposure to 5 mL of 0.2 mg/L amidosulfuron per plant resulted in no reduction of mature plant height but caused a delay in flowering of about 1–2 days, as in our previous study (Yu et al., 2009). However, due to a need to select suitable amidosulfuon dose to balance the gametocidal effect on the anther and other phytotoxic effects on the pistil and vegetation organs, some side effects that were showed as chlorosis on young leaf (Figure 1A), short retard of raceme elongation, and increase of anthocyanin (Figure 1B) in 2–8 DAT were observed in all the treated plants. Excess exposure (3 μg per plant or higher dose) would inhibit the plant growth and depigment the petal (Figure 1B). The filaments of the 0.2 mg/L amidosulfuron treated plants were much shorter and the anthers were thinner than that of the control (Figure 1C). Complete male sterility in rapeseed plants could be easily achieved by amidosulfuron treatment. The pollen grains of sterile anthers in the treated plants were unviable, without aceto-carmine staining (Figure 1D). In addition, we made a postponed spraying to the day when the first flower was opening. The treated plants could maintained fertile phase for about 7–9 days and then the subsequent flowers, whose anther development was at the stage of vacuolated microspore when they were subjected to the treatment, converted to male sterile state. This suggested that amidosulfuron mainly restrain the pollen development before microspore mitosis. The morphological changes on the stem elongation, leaf color, and flower traits suggested phytotoxic effect of the treatment on cell growth, chloroplast structure, and flavonoid biosynthesis.

### Effect of amidosulfuron treatment on chloroplast structure, photosynthesis, and energy metabolite

**Figure 2:**
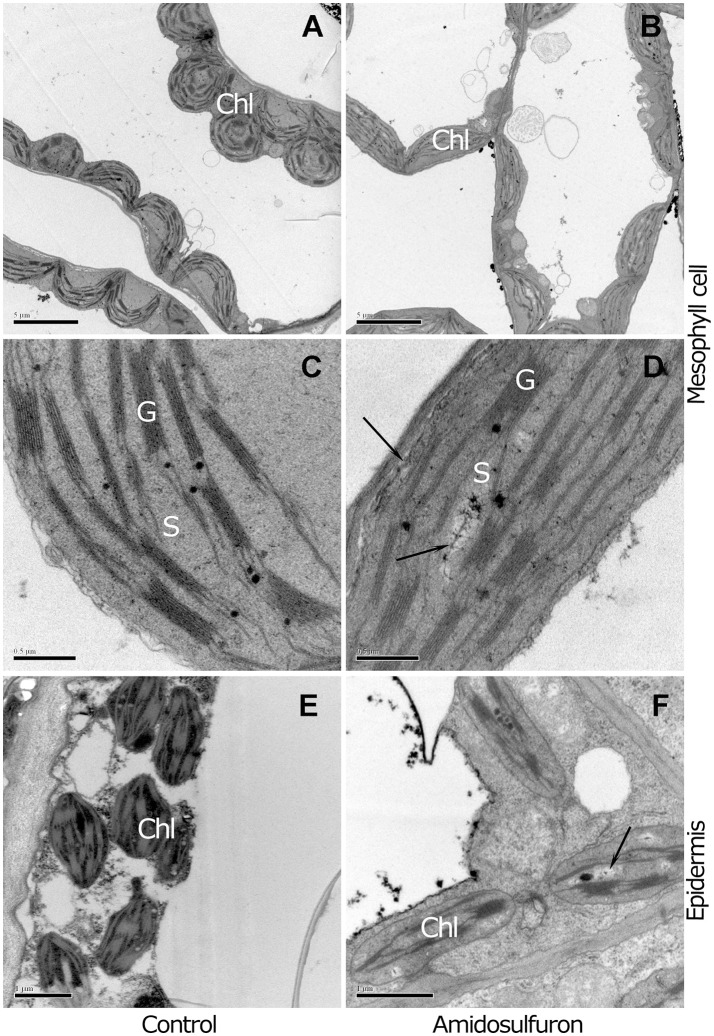
Comparison between mesophyll and epidermal chloroplasts of the control and treated plants. **(A)** The mesophyll cell chloroplasts of the control contain numerous grana, stacks of appressed thylakoid membranes. **(B)** In contrast to the plump chloroplasts in the control, the chloroplasts in the treated plants are flat and undeveloped. **(C)** The magnified structures show electron-dense lipid droplets known as plastoglobuli accumulate. **(D)** Flat chloroplasts with undeveloped thylakoid membrane and destructed zone pale in color (Arrowhead). **(E)** Chloroplasts in anther epidermis of the control contain intrinsic thylakoid membranes and starch granule. **(F)** The treated plants show a distinctive appearance, with loss of grana stacks and few plastoglobuli. Chl, chloroplast; G, grana thylakoids; S, stroma. Scale bar = 5 μm **(A,B)**, 0.5 μm **(C,D)**, and 1 μm **(E,F)**.

Since the symptom of young leaf chlorosis suggested that the amidosulfuron treatment could affect the photosynthetic capability, we also paid attention on the chloroplast structure in the anther and leaf. The normal chloroplasts in leaf mesophyll cell were almost spherical and contained numerous grana and stacks of thylakoid membranes, (Figures 2A,C). In contrast, the amidosulfuron-treated mesophyll chloroplasts were generally flat with necrotic cavities and undeveloped grana (Figures 2B,D). These results suggests that amidosulfuron treated plants may have less photosynthetic capacity than control plants. Moreover, the chloroplasts in epidermal cells of the treated plants lost thylakoid membrane, grana stacks and other components (as in Figure 2E), giving the tissues a distinctive appearance (Figure 2F). The chloroplasts in leaf, epidermis, and endothecium of the anther were obviously destructed by the amidosulfuron treatment.

**Figure 3:**
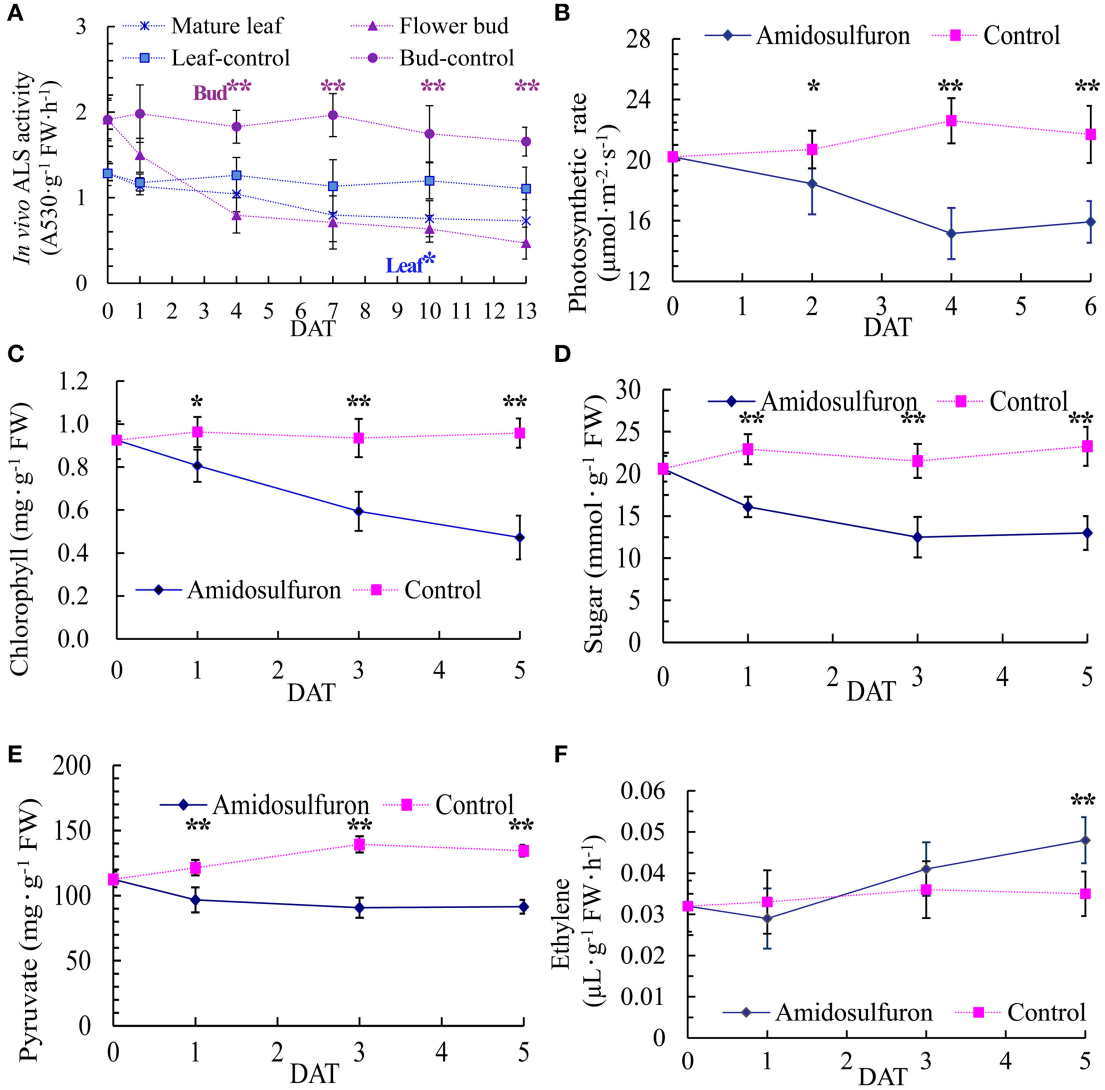
Effect of amidosulfuron on *in vivo* ALS activity, photosynthetic rate, the amount of leaf chlorophyll, soluble sugar, pyruvate, and ethylene release rate in the flower buds several days after amidosulfuron treatment (DAT). **(A)** *In vivo* ALS activity. **(B)** Photosynthesis rate. **(C)** Content of chlorophyll. **(D)** Content of soluble sugar. **(E)** Content of pyruvate. **(F)** Release rate of ethylene. Error bars represent ± one standard deviation (*n* = 3). ^*^ and ^**^ indicate significant difference at confidential level of 0.05 and 0.01 by Student's *t*-test.

Because ALS is the direct target of sulfonylurea herbicides, 0.2 mg/L amidosulfuron exposure obviously inhibited the *in vivo* ALS activity in leaf and flower bud (Figure 3A). The ALS activity of flower bud was higher than mature leaf but decreased more after the exposure. Leaf photosynthesis showed a severe descent in amidosulfuron treated plants (Figure 3B) detected by using the Li-6400 portable photosynthetic system. Moreover, application of amidosulfuron also reduced the content of leaf chlorophyll, pyruvate, and soluble sugar (Figures 3C–E) within the several days after treatment. Thus, we assumed that the destruction of chloroplast and incapability of photosynthesis were likely to be a major physiological effect of the amidosulfuron treatment at gametocidal dose. In addition, ethylene release rate in the flower bud samples after amidosulfuron treatment had a slightly decreased on 1 DAT and then increased significantly on 3 and 5 DAT (Figure 3F).

### Changes in free amino acid pool and BCAA content

**Figure 4:**
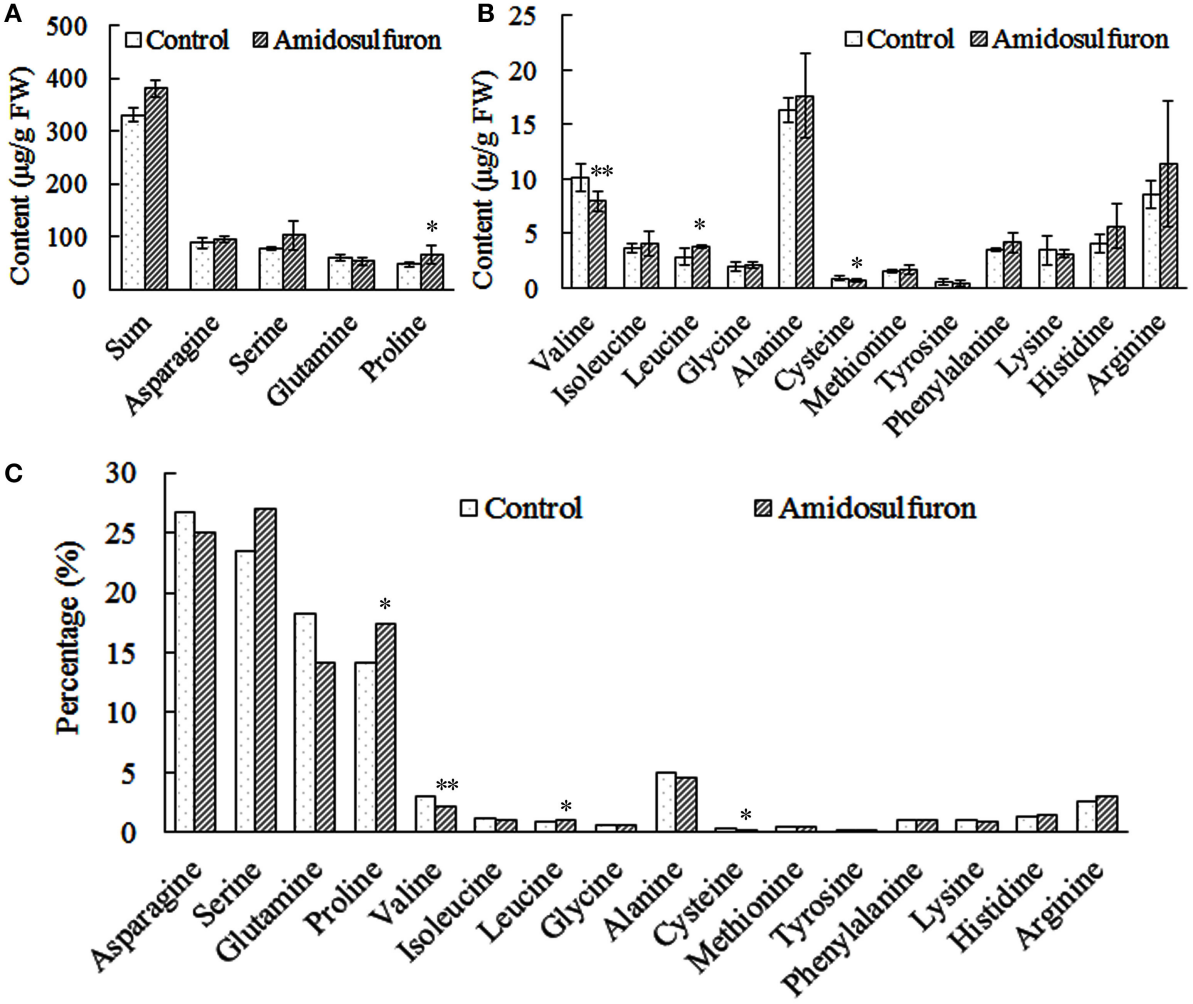
The content and percentage of 16 free amino acids in the anthers of the control and amidosulfuron treatment. Error bars represent ± one standard deviation (*n* = 3). ^*^ and ^**^ symbols indicate the value is significant at 90 and 95% confidential level, respectively. **(A)** The sum of 16 amino acids and the content of four most abundant amino acids. **(B)** The content of three branched-chain amino acids and the others. **(C)** The percentage of 16 amino acids in the total free amino acids pool.

The content of 16 kinds of free amino acids except threonine (Figure 4) could be detected in the anthers of the treated plants and the corresponding control. The amidosulfuron treatment led to a slight rise (15.11%) of total free amino acid and an obvious change of the proportions of some components (Figure 4). Surprisingly, the total amount of the three BCAAs was reduced only by 4.98%, valine decreased by 21.40% but isoleucine and leucine increased by 11.33 and 32.64%, respectively. In addition, the content of cysteine declined while proline, which has long been regarded as pivotal amino acids for male sterility, ascended significantly. To sum up, the amidosulfuron treatment did not significantly deprive BCAAs as previous study assumed (Ray, 1984), but change the proportions of several components in the free amino acid pool.

### Cytological characteristics of male sterility induced by amidosulfuron

**Figure 5:**
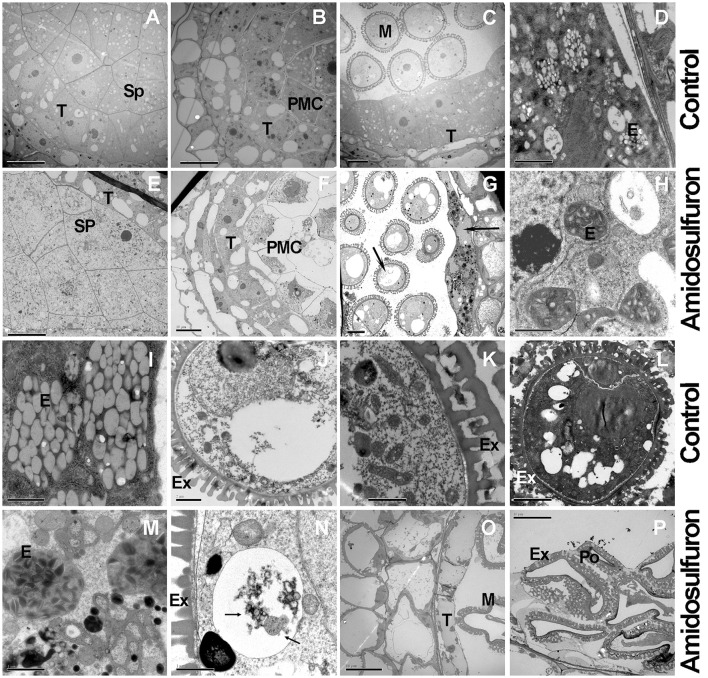
Transmission electron micrographs of cross-sections through anther of the amidosulfuron-treated plants and the control. At uni-nucleate microspore stage, autolysosomes or large vacuoles form in tapetum and microspore of the treated plants. Then tapetum and microspore breakdown quickly. **(A)** The fertile anthers at sporogenous cell (Sp) stage. **(B)** Pollen mother cells in the fertile anther. **(C)** Uni-nucleate microspore and tapetum in the fertile anther. **(D)** Plentiful juvenile elaioplasts (derived from plastid) in condensed cytoplasm. **(E)** Sporogenous cell in the treated plants. **(F)** Tapetum with large cytoplasmic vacuoles in the treated plants. **(G)** At uni-nucleate microspore stage, premature breakdown begin and large vacuoles (Arrowheads) containing autophagosomes form in tapetum and microspore. **(H)** Malformed elaioplasts in the treatment. **(I)** Elaioplasts in the normal tapetum, full with nonosmiophilic globular. **(J)** Vacuolated microspore in normal plants. **(K)** Fertile pollen contains plentiful organelle inside, with exine outside. **(L)** Fertile pollen full with organelle and osmiophilic compounds. **(M)** The degraded tapetum showing elaioplasts and tapetosomes and accumulated lipid. **(N)** Vacuolar or autophagic cell death (Arrowheads) in the microspores of the treated plants. **(O)** Degenerated tapetum and pollen. **(P)** Aborted pollen in the treated anthers. E, elaioplast; Ex, exine; M, microspore; PMC, pollen mother cell; Po, pollen grain. Sp, sporogenous cell; T, tapetum; Scale bars = 10 μm **(A-C,E-G,L,O,P)** and 2 μm **(D,H–K,M,N)**.

Microscopic studies revealed that the pollen abortion in amidosulfuron treated plants was associated with abnormal behaviors both in the tapetal cells and PMCs with additional defects found in both compartments at the later microspore stages. Normal tapetal cells should contain abundant organelles such as the nucleus, vacuoles, vesicles, endoplasmic reticulum, and plastids (Figures 5A–D). In the treated plants (Figures 5E–H), however, both PMCs and tapetal cells were damaged and showed shriveled nucleus and cytoplasm (Figure 5F). The number of organelles decreased and a lot of large vacuoles containing autophagosomes formed in tapetum and in the microspore (Figure 5G, Arrowhead). Degeneration of plastid, mitochondria, and phagocytosis were observed both in the microspore and tapetal cell of the treated plants (Figures 5G,H,M). At the mid microspore stage, the structure of elaioplast in the treated plants was obscure and some autophagic vacuole formed in tapetal cell (Figure 5H). By the vacuolated microspore stage, the tapetal cells should contain large number of elaioplasts, which were showed as plastoglobuli that contain nonosmiophilic bodies (Figure 5I). However, in the treated plants, the tapetum was disintegrated and the elaioplasts were stained deeply, with malformed structure (Figure 5M). The pollen grains in the control plant had well-formed organelles and pollen walls (Figures 5J–L). In the aborted microspores, cytoplasm was degraded by autophagic vacuoles, producing large vacuoles (Figure 5N). Then the microspore cytoplasm in the treated plant was completely degraded (Figures 5O,P). In addition, exine was thickened but poorly differentiated (Figures 5N,P). These evidenced aberrant lipid metabolism and transportation. In one word, the amidosulfuron treatment resulted in autophagy in the tapetal cells and microspores at early development stages and consequently, defective elaioplasts, and pollen exine.

### Analyses of differential gene expression in response to ALS-inhibiting herbicides

**Figure 6:**
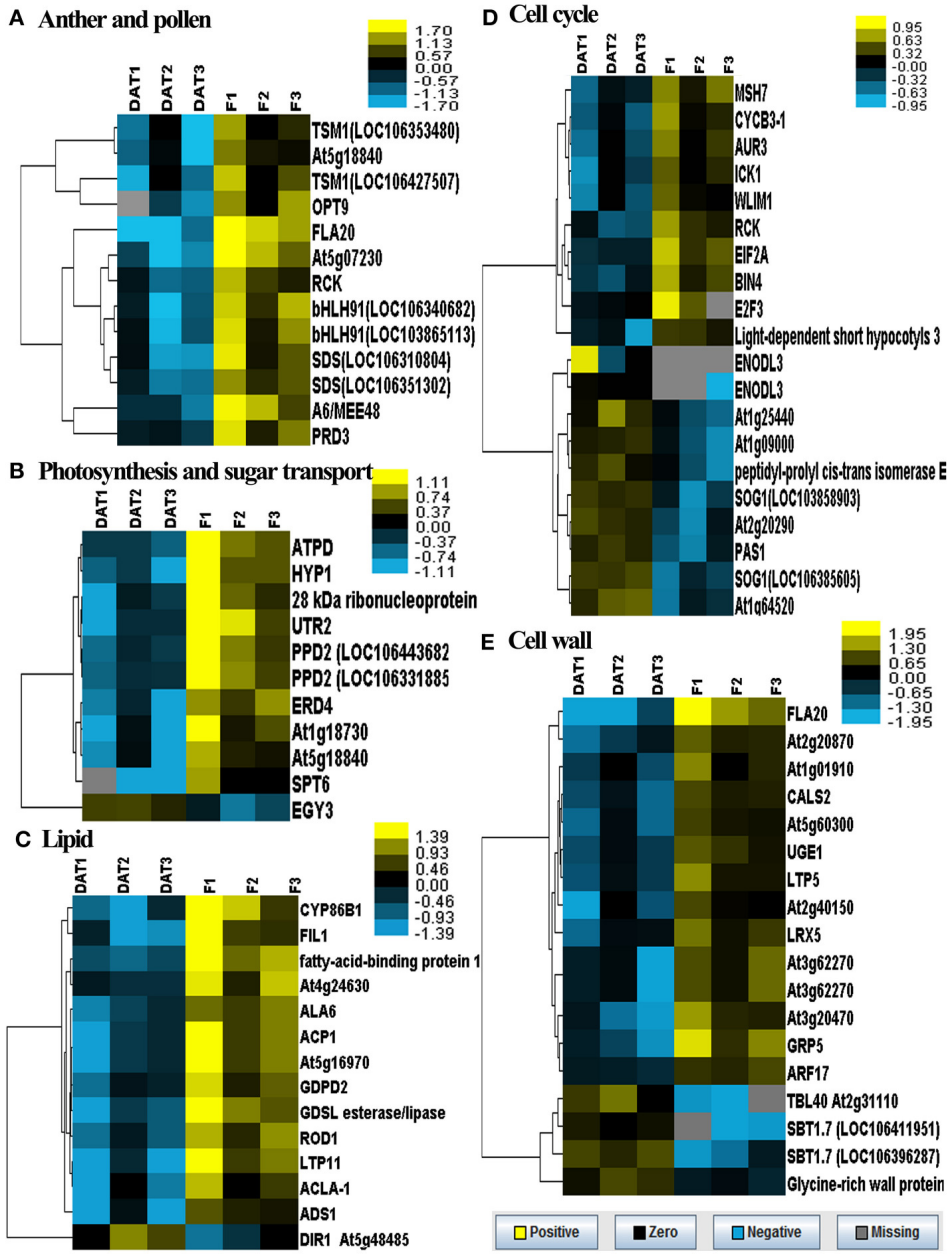
Heatmaps of log-transformed expression level of five groups of selected DETs in the two groups of flower bud samples. DAT1, DAT2, and DAT3 represent the replicates of amidosulfuron treatment and F1, F2, and F3 represent the replicates of control. **(A)** Genes related to anther and pollen development. **(B)** Genes related to photosynthesis and sugar transport. **(C)** Genes related to lipid metabolism. **(D)** Genes related to cell cycle. **(E)** Genes related to cell wall construction. The detailed gene information was listed in Data Sheet S1, highlighted with yellow background color.

In order to profile the DGE tags, we sequenced two groups of cDNA libraries of young flower buds: group DAT for amidosulfuron-treated plants and group F for the control plants, on Illumina Solexa sequencing platform. We obtained 7,911,523 and 9,189,278 clean reads (under GEO accession GSE69679), respectively, after a raw tags quality filter. The clean reads were mapped based on *B. napus* genome information in JGI and NCBI databases, as well as a previous rapeseed flower bud transcriptome assembly (unpublished), and 105,220 and 108,161 transcripts were identified in the treatment and the control, respectively. To compare differential expression patterns among the two groups of libraries, we normalized the tags distribution for gene expression level in each library to extract significant DETs. Totally, 193 up-regulated and 283 down-regulated DETs (Data Sheet S1) were extracted, out of which 142 and 201 transcripts, respectively, were functionally annotated by BLAST search in NCBI database (Data Sheet S1). The 343 annotated DETs could be assigned in to dozens of categories (Table 1), in which important categories included meiosis, cell division, cell wall formation, cytoskeleton, gametophyte, chloroplast, RNA processing, DNA replication, protein synthesis and degradation, lipid metabolism, oxidoreductase, defense response, detoxification, and so on. The expression levels of five groups of DETs in the six samples were showed by heatmaps (Figure 6). Most genes belonging to the groups of anther and pollen development, cell wall construction, lipid metabolism, photosynthesis and sugar transport, and some key genes in the group of cell division and growth were down-regulated.

**Table 1:** Functional classification of differentially expressed transcripts (DETs).

**Category**	**Up-regulated**	**Category**	**Down-regulated**
Meiosis	4	Meiosis	6
Cell division	9	Cell division	8
Cell expansion	1	Cell expansion and growth	5
Cell wall	8	Cell wall	13
Cytoskeleton	1	Cytoskeleton	1
Gametophyte	1	Tapetum	4
Embryogenesis and megagametophyte	5	Gametophyte	2
Pollen tube	2	Embryogenesis and megagametophyte	1
Chloroplast	1	Pollen tube	1
Ribosome	14	Photosynthesis	5
RNA processing	14	Chloroplast	17
DNA replication	2	Mitochondria	6
Purine metabolism	3	RNA processing	3
Chromatin	2	DNA replication	1
tRNA ligase	5	Pyrimidine	1
Transcription and regulation	19	Transcription and regulation	3
Protein synthesis	2	Protein synthesis	2
Protein modification and degradation	25	Protein modification and degradation	10
Fatty acid and lipid	2	Chaperone	3
Transport	7	Fatty acid and lipid	14
Translocation	2	Carbohydrate	2
Defense response	6	Xlyoglucan degradation	2
Oxidoreductase	1	Transport	4
Detoxification	1	Sugar transport	4
		Amino acid	2
		Signal	3
		Defense response	20
		Oxidoreductase	4
		Hormone response	3
		Secondary metabolite	11
		Glucosinolates degradation	4
		Lignin	3

**Figure 7:**
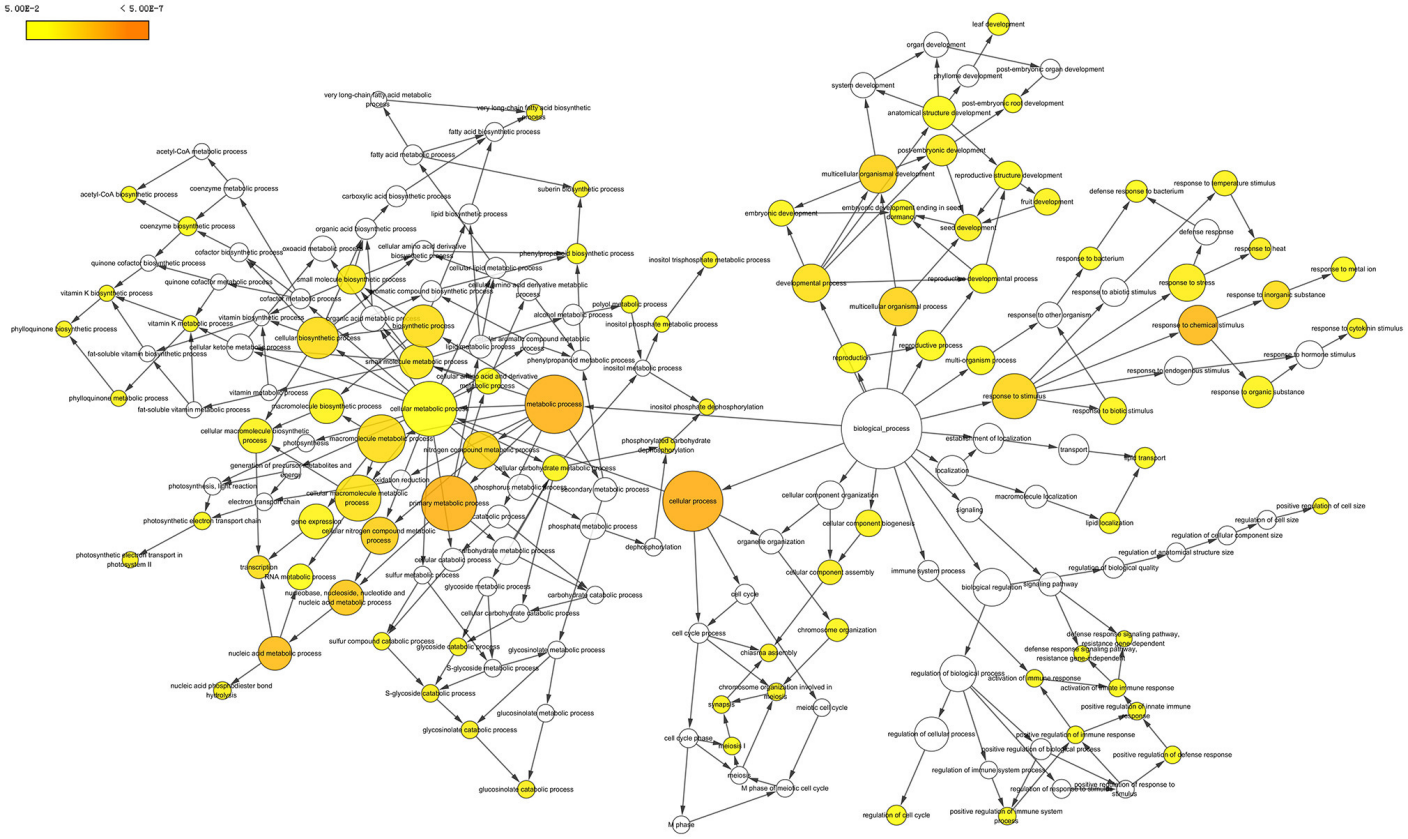
Gene ontology network enriched based on the differentially expressed transcripts. The diagram was produced by BiNGO in Cytoscape software. Yellow color in a node indicated the significant level.

We searched those DETs for GO terms and 357 GOs were significantly enriched by BiNGO program with hypergeometric test (Figure 7 and Data Sheet S2). The network in Figure 7 showed that GO of defense response, cell division, and regulation of flower development were the main disturbed genes by the amidosulfuron treatment on the flower buds transcriptome. Furthermore, we searched the GO terms corresponding to nine major categories (Table 2) and found the expression of 4, 33, 40, 30, 56, 33, 8, 16, and 11 genes were affected, respectively in amino acid metabolism, energy metabolism, metabolism of carbohydrate, lipid and protein, cell division and death, defense response, chlorophyll and mitochondria, hormone, oxidoreduction, and phenylpropanoid biosynthesis. A gene encoding ethylene-responsive transcription factor RAP2-11-like was up-regulated, meanwhile, a gene in auxin mediated signaling pathway (GO:0009734) was down-regulated. The effect on ethylene mediated signaling pathway was supported by assay of ethylene release rate (Figure 3F). Four GO terms belong to protein disulfide isomerase, protein disulfide oxidoreductase activity, protein folding, glycosylation, homodimerization activity were up-regulated. This intimated an occurrence of strong protein modifications due to amidosulfuron application.

**Table 2:** GO enrichment for nine important categories.

**Category**	GO terms^*^	**Up-regulated genes**	**Down-regulated genes**
Amino acid metabolism	GO:0006564; GO:0004813; GO:0006419; GO:0004828; GO:0006434; GO:0004813; GO:0016597	3	1
Energy metabolism	GO:0005524; GO:0006754; GO:0003878; GO:0015986; GO:0004022; GO:0008106	17	16
Carbohydrate, lipid, protein metabolism	GO:0019252; GO:0005529; GO:0003978; GO:0008289; GO:0016042; GO:0006629; GO:0006869; GO:0005515; GO:0046983; GO:0003756; GO:0015035; GO:0006457; GO:0006486; GO:0042803; GO:0004672; GO:0019901; GO:0004674; GO:0015031; GO:0006508; GO:0004185; GO:0004252	7	33
Cell division, growth, and death	GO: 0048443; GO: 0006075; GO: 0010228; GO: 0030307; GO: 0045793; GO: 0045740; GO: 0009911; GO: 0048366; GO: 0000082; GO: 0051026; GO: 0007050; GO: 0008219; GO: 0051301; GO: 0045454; GO: 0005618; GO: 0052325; GO: 0007047; GO: 0051276; GO: 0000775; GO: 0008360; GO: 0045995; GO: 0040020; GO: 0006914; GO: 0006915	2	28
Stimulus and defense response	GO: 0009627; GO: 0019137; GO: 0071475; GO: 0043623; GO: 0071215; GO: 0070301; GO: 0034599; GO: 0016036; GO: 0042631; GO: 0006952; GO: 0042742; GO: 0050832; GO: 0009737; GO: 0009733; GO: 0009617; GO: 0080021; GO: 0009607; GO: 0046686; GO: 0010200; GO: 0009409; GO: 0046898; GO: 0034976; GO: 0010332; GO: 0009408; GO: 0042542; GO: 0080167; GO: 0009416; GO: 0009624; GO: 0010167; GO: 0006979; GO: 0010193; GO: 0000302; GO: 0009751; GO: 0009651; GO: 0006950; GO: 0009636; GO: 0009414; GO: 0010043	14	42
Chlorophyll and mitochondria	GO: 0015979; GO: 0015995; GO: 0009507; GO: 0009941; GO: 0009570; GO: 0009543; GO: 0009535; GO: 0005743; GO: 0005759; GO: 0005746; GO: 0005739	15	18
Hormone	GO: 0009873; GO: 0009734; GO: 0009867; GO: 0080024; GO: 0080032; GO: 0080031; GO: 0009697	3	5
Oxidoreductase	GO: 0016491; GO: 0016705; GO: 0016706; GO: 0016717; GO: 0016702; GO: 0019825; GO: 0009654; GO: 0015671; GO: 0004601; GO: 0005782	3	13
Phenylpropanoid biosynthesis	GO: 0043481; GO: 0016117; GO: 0047763; GO: 0042409; GO: 0051555; GO: 0045431; GO: 003379; GO: 0045486	0	11

* *The details of GO terms can be find in Gene Ontology Consortium (http://www.geneontology.org/)*.

Surprisingly, the pathway of BCAAs synthesis by ALS enzyme showed no obvious change. Otherwise, a few of amino acid metabolic processes were affected by the treatment, but at a lower stringency level. Four GOs including alanine-tRNA ligase activity (GO:0004813), serine-tRNA ligase activity (GO:0004828), alanyl-tRNA aminoacylation (GO:0006419), and seryl-tRNA aminoacylation (GO:0006434) were up-regulated but serine biosynthesis (GO:0006564) was down-regulated. This was similar to the results of another study (Das et al., 2010) that except threonine ammonia lyase and six genes related to other amino acid biosynthesis, the expression of most genes related to the BCCA group was not changed.

**Figure 8:**
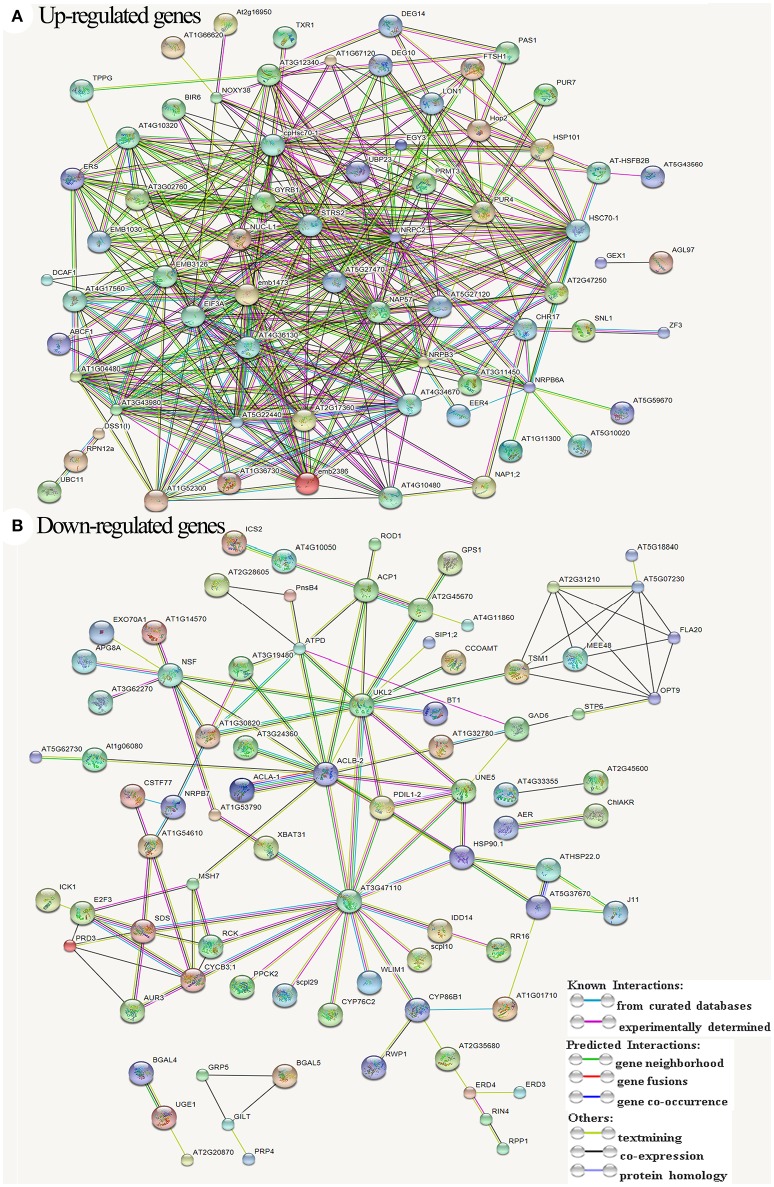
All known and predicted protein-protein interactions based on STRING analysis of Arabidopsis genes homologous to the differentially expressed transcripts. **(A)** Interactions among the up-regulated genes. **(B)** Interactions among the down-regulated genes. The symbols and names of all proteins/genes were listed in Data Sheet S1.

We further showed the possible protein-protein interactions among the DETs by a network (Figure 8) produced by STRING. It was showed that some proteins may play important roles in this regulation network, in which glucan endo-1,3-beta-glucosidase A6 (MEE48), fasciclin-like arabinogalactan protein 20 (FLA20), oligopeptide transporter OPT9, caffeoyl-CoA 3-O-methyltransferase (TSM1), cyclin-SDS, ATP-dependent DNA helicase homolog HFM1 (RCK), DNA mismatch repair protein MSH7, putative recombination initiation defects PAIR1 (PRD3), and putative cyclin B3-1 (CycB3;1) were involved in meiosis and formation of pollen cell wall. Some other important genes that were up-regulated in the network encode several EMB (embryogenesis) family proteins, ribosomal proteins, serine-tRNA ligase At5g27470, mitochondrial protease homolog LON1, putative Do-like protease DEG14 and DEG10, and NUC-L1 that involved in pre-rRNA processing and ribosome assembly.

There were 32 KEGG biological pathways (Table 3) affected by the treatment, mainly including aminoacyl-tRNA biosynthesis, amino acid metabolism, cytochrome P450 mediated drug metabolism, benzoate degradation via CoA ligation, purine and pyrimidine metabolism, RNA polymerase, fatty acid metabolism, porphyrin and chlorophyll metabolism, photosynthesis, glycolysis/gluconeogenesis, oxidative phosphorylation, citrate cycle, CO_2_ fixation, and biosynthesis of secondary metabolites (steroid, terpenoid, ubiquinone, and so on). The results showed that the expression of most DETs in cell division and growth, energy metabolite, and flavonoid biosynthesis were inhibited. The altered expression of genes related to chlorophyll, chloroplast, and mitochondria indicated the abnormal development in chloroplast and mitochondria, in line with our cytological observation.

**Table 3:** KEGG biological pathways altered by amidosulfuron treatment.

**Pathway ID**	**Description**	**Significant gene**	**Tested gene**	***P*-value**
00970	Aminoacyl-tRNA biosynthesis	4	158	0.0021
00903	Limonene and pinene degradation	3	96	0.0046
00720	Reductive carboxylate cycle (CO_2_) fixation	2	48	0.0128
00230	Purine metabolism	4	292	0.0178
00626	Naphthalene and anthracene degradation	2	62	0.0205
00361	gamma-Hexachlorocyclohexane degradation	2	66	0.0230
00945	Stilbenoid, diarylheptanoid, and gingerol biosynthesis	2	72	0.0269
03020	RNA polymerase	2	73	0.0276
04623	Cytosolic DNA-sensing pathway	1	8	0.0309
00240	Pyrimidine metabolism	3	218	0.0399
00624	1- and 2-Methylnaphthalene degradation	1	14	0.0510
00980	Metabolism of xenobiotics by cytochrome P450	1	17	0.0609
01053	Biosynthesis of siderophore group nonribosomal peptides	1	17	0.0609
00982	Drug metabolism—cytochrome P450	1	19	0.0675
00020	Citrate cycle (TCA cycle)	2	145	0.0918
00632	Benzoate degradation via CoA ligation	1	34	0.1152
00100	Steroid biosynthesis	1	42	0.1396
00410	beta-Alanine metabolism	1	51	0.1664
01040	Biosynthesis of unsaturated fatty acids	1	53	0.1722
00900	Terpenoid backbone biosynthesis	1	55	0.1780
00350	Tyrosine metabolism	1	57	0.1838
00052	Galactose metabolism	1	70	0.2203
00071	Fatty acid metabolism	1	80	0.2473
00130	Ubiquinone and other terpenoid-quinone biosynthesis	1	80	0.2473
00860	Porphyrin and chlorophyll metabolism	1	84	0.2579
00650	Butanoate metabolism	1	88	0.2683
00260	Glycine, serine, and threonine metabolism	1	120	0.3467
00250	Alanine, aspartate, and glutamate metabolism	1	120	0.3467
00195	Photosynthesis	1	133	0.3762
00010	Glycolysis/Gluconeogenesis	1	190	0.4912
00520	Amino sugar and nucleotide sugar metabolism	1	208	0.5231
00190	Oxidative phosphorylation	1	396	0.7601

### Expression analysis of *ALS* and other important genes detected by real-time PCR

**Figure 9:**
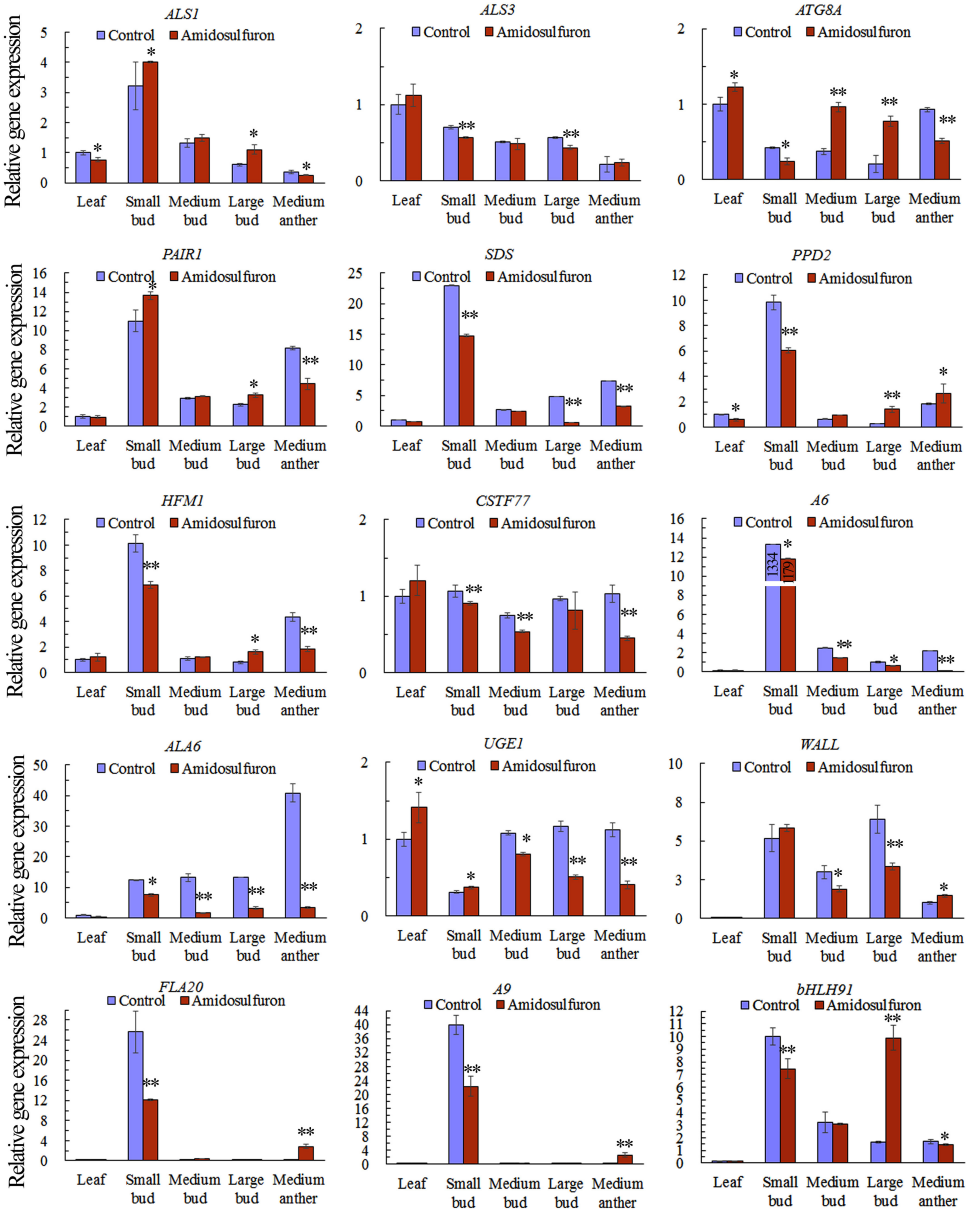
Relative gene expression in the indicated tissues of *ALS* genes and 13 differentially expressed transcripts (*ATG8A, PAIR1, SDS, PPD2, HFM1, CSTF77, A6, ALA6, UGE1, Wall, FLA20, A9*, and *bHLH91*). The y-axes represent the normalized relative gene expression level, which is the −2^ΔCt^ value of qRT-PCR compared to the internal control gene *BnActin7*. The details of the 13 selected genes are included in Data Sheet S1, highlighted in yellow and written in red colors. The accession numbers for 13 DETs are also in Table S1. ^*^ and ^**^ indicate significant difference at confidential level of 0.05 and 0.01 by Student's *t*-test. Error bars represent ± one standard deviation (*n* = 3).

We found that the expression of *ALS1* and *ALS3*, detected via qRT-PCR, were not affected greatly in the treated plants because the differences among the treatment and control were less than one-fold (Figure 9) though significant changes estimated by Student's *t*-test were found in some organs. This suggested that amidosulfuron treatment at gametocidal dose did not greatly disturb the *ALS* expression although the enzyme activity were obviously inhibited as above mentioned. The other 13 interesting DETs, which were showed in Figure 6, were also chosen for gene expression analysis. The results showed that, in the small buds (1 mm long), the relative expression levels of all selected DETs were in line with the DGE profiling data, though expression patterns for these genes changed with different size of flower buds and anthers (Figure 9). Three genes that encode DNA helicase HFM1, cyclin-SDS-like, and PAIR 1-like (homologous pairing aberration in rice meiosis 1), respectively, which are important for cell cycle control, and three genes that encode glucanase A6, putative cell wall protein LOC106368794, and bifunctional UDP-glucose 4-epimerase/UDP-xylose 4-epimerase (UGE1), which are involved in microspore release from tetrad and cell wall formation, were down-regulated. We also found that *CSTF77* (*cleavage stimulation factor subunit 77*), chloroplastic *PPD2* (*psbP domain-containing protein 2*), *ALA6* (*phospholipid-transporting ATPase 6*), and *FLA20* were depressed in the amidosulfuron treatment. Two tapetum-specific genes *A9* and *bHLH91* were also down-regulated in the small flower buds of the treated plants. Notably, an important gene *ATG8A* encoding autophagy-related protein 8a was up-regulated by amidosulfuron treatment.

## Discussion

### Destruction of plant photosynthetic system is associated with male sterility induced by ALS-inhibitors

Indubitably, ALS is the direct target of various sulfonylurea herbicides (McCourt et al., 2006; Zhou et al., 2007). In the present study, even trace amount of amidosulfuron can significantly inhibit the ALS activity in rapeseed flower buds. Constitutive or anther-specific expression of ALS mutant *csr1-1D* reversed the male sterile phenotype induced by tribenuron-methyl in both rapeseed and *Arabidopsis* (Zhao et al., 2015). Although, plant ALS are encoded by nuclear genes, their proteins are located in plastid and chloroplast (Shimizu et al., 2008). Sulfonylurea derivatives do not directly inhibit the process of photosynthesis but their activity may impair the efficiency of the photosynthetic apparatus (Saja et al., 2016). We found that the amidosulfuron treatment did not severely affect plant growth but it diminished the photosynthetic rate and chlorophyll content significantly. Tribenuron-methyl and other ALS-inhibiting gametocides had similar effects to amidosulfuron, when a sub-herbicidal dose was used (data not showed). Abnormality of chloroplast and tapetal elaioplast caused by sulfonylurea gametocides was firstly reported in a dissertation (Yu, 2009). Zabalza et al. (2013) found that carbohydrates accumulated in the leave and roots of pea plants following treatments with both inhibitors of KARI and ALS. Both types of inhibitors induce growth arrest and photosynthesis inhibition (Zabalza et al., 2013). However, they assumed that carbohydrate accumulation in the leaf occurred as a consequence of a decrease in sink strength. In another study, exposure of *A. thaliana* plants to trace concentration of imazethapyr strongly affected chlorophyll synthesis and increased reactive oxygen species (ROS) (Qian et al., 2011, 2015). Short exposure of rice seedlings to imazethapyr induced damages to lipid membranes and negatively affected the transcription of a number of genes involved photosynthesis, starch and sugar metabolism, and the tricarboxylic acid cycle (Qian et al., 2011; Sun et al., 2016). Cheng et al. (2013) and Li et al. (2015) also found that, at the vacuolated-microspore stage of MES-treated rapeseed anther, the chloroplasts in the epidermis and endothecium cells exhibited defects. Both amidosulfuron (Figure 3) and MES exposure (Li et al., 2015) led to decrease in soluble sugars content in leaf and flower bud, and therefore, it was assumed that carbohydrate metabolism maybe blocked in these treatments. Moreover, the results of Sun et al. (2016) showed that *A. thaliana* cultivated in a solution containing 20 μg/L imazethapyr produced more anthocyanins and ROS, particularly in the *pgr5* mutant (a defect in the proton gradient regulation 5 pathway). Their results also showed that the PSII was severely damaged and the transcript levels of most photosynthesis-related genes were decreased in the treated plants (Sun et al., 2016).

In summary, destruction of chloroplast and incapability of photosynthesis are two main effects observed in the treatments of sulfonylurea and other ALS-inhibiting gametocides (Qian et al., 2011; Cheng et al., 2013; Li et al., 2015; Saja et al., 2016; Sun et al., 2016). Moreover, the down-regulation of chloroplastic *PPD2* intimated structural and/or functional abnormalities of chloroplast. Thus, we assumed that amidosulfuron will affect the chloroplast and photosynthesis as it enters the plant body. Developing microspores constitute a strong photosynthetic sink and accumulate photoassimilates (De Storme and Geelen, 2014), such as starch and other carbohydrates, and thus the photosynthesis and carbohydrate supply is very crucial for microsporogenesis. Deprivation of the anther sugar content is often considered one of the predominant factors underlying stress-induced male sterility (Reviewed by De Storme and Geelen, 2014). Thus, we assumed that deprivation of photosynthesis and chlorophyll by ALS-inhibitors also contribute to inducing male sterility. However, how ALS-inhibitors affect chloroplast structure and function need further investigation.

### Cytological abnormalities of gametocide-induced male sterility

Tapetum plays crucial roles in microspore development and hence was often affected by various abiotic stresses including some chemicals (Reviewed by De Storme and Geelen, 2014). In benzotriazole and ethrel treated *B. juncea* anthers, tapetum exhibited premature degeneration of mitochondria and plastids (Singh and Chauhan, 2007). It was reported that pollen abortion in benotrazole-treated sunflower was mainly associated with abnormal behavior of tapetum (Tripathi and Singh, 2008). A limited number of anther locule showed early degeneration of tapetum followed by disintegration of sporogenous tissues (Tripathi and Singh, 2008). This phenomenon was similar to the results of Cheng et al. (2013), Li et al. (2015), and the present study. Zhao et al. (2015) revealed that autophagy was elevated in rapeseed anther by both tribenuron-methyl treatment and anther-specific knockdown of *ALS*. We also found that tissue autophagy occurred at the early stage of microsporogenesis in the anther of amidosulfuron treated plants. Taken together, these results suggested that tapetum development and function were affected by various gametocides.

Callose wall formation and dissolution is a unique feature of male meiosis in plants during and after meiotic division (Zhang et al., 2007). The wall covering PMC and tetrad microspore is composed mainly of β-1, 3-glucans, and pectin. β-1,3-glucanase is the main enzyme responsible for callose wall degradation and release individual microspores at the end of meiosis. Recent research has suggested that the expression of *glucan endo-1, 3-beta-glucosidase A6* was tapetal specific and necessary for *Arabidopsis* pollen development (Zhang et al., 2007). The down-regulation of tapetum marker gene *glucanase A6* in the amidosulfuron treated plant suggested that the callose layer covering PMC and tetrad microspores should be affected. Down-regulation of the other genes involved in cell wall formation (Figure 6) also implied the abnormalities on pollen wall construction.

### Change of free amino acid metabolism

Some early studies about mode of action for ALS-inhibiting herbicides focused on the influences on BCAAs synthesis or protein metabolism (Ray, 1984). BCAAs starvation was thought to be responsible for the phytotoxic effect. Zhao et al. (2015) also reported more tribenuron-methyl accumulation and subsequent stronger ALS inhibition and BCAAs starvation in anther than in leaf and stem. However, other researches elicited some conflicts with the hypothesis deduced by the earlier studies (Zhou et al., 2007). Valine and isoleucine were ineffective in preventing the damage induced by chlorsulfuron on the plasma membrane of maize roots (Giardina et al., 1987). In addition, growth inhibition in seedlings of corn and pea due to chlorsulfuron treatment was not alleviated by valine and isoleucine (Giardina et al., 1987). Shaner and Singh (1993) found that application of ALS inhibitor imazaquin, KARI inhibitors Hoe704 in maize led to an increase of total free-amino acid. Leucine was also rising while the isoleucine, valine, and 2-aminobutyric acid (amino acid formation of ALS substrate butanone) dropped. Höfgen et al. (1995) reported that in transgenic potato with antisense *ALS* gene, total free amino acid and content of BCAAs increase significantly. Increase of total free amino acids after sulfonylurea treatments was also reported in some other studies (Royuela et al., 2000; Shim et al., 2003). Once again, our present results suggested that among three BCAAs, only valine content dropped but isoleucine and leucine rose (Figure 4). It is possible that the amidosulfuron treatment led to an increase of protein turn-over (disintegrated protein become amino acid again), and hence more amino acids were released into free amino acid pool (Royuela et al., 2000). Thus, the amidosulfuron treatment did not significantly deprive BCAAs as other herbicide did in the previous study (Ray, 1984), but change the component proportions of free amino acid pool. This would implicate a systematical sexual dysfunction caused by amino acid imbalance.

Surprisingly, the expression of *ALS* and it's relatives did not greatly changed but hundreds of non-target-site transcripts were altered by the amidosulfuron treatment, consistent with the literatures about other ALS-inhibiting herbicides (Das et al., 2010; Doğramacı et al., 2015; Duhoux et al., 2015; Li et al., 2015). We assumed that protein turn-over and imbalance of free amino acid pool might offset the minor shortage of BCAAs. Thus, we could again draw a conclusion that BCAAs starvation maybe not the main reason for stamen abortion induced by gametocide amidosulfuron.

### Ethylene releasing indicated cell cyclemaybe inhibited by amidosulfuron exposure

As a major plant hormone, ethylene regulates some biological pathways related to flower senescence and cell death (Rogers, 2013). Application of imazameth, metsulfuronmethyl, or prosulfuron on citrus fruit increased the levels of internal ethylene (Burns et al., 1999). Ethylene release rate in the flower bud samples after amidosulfuron treatment increased significantly on 3 and 5 DAT (Figure 3F). A gene encoding ethylene-responsive transcription factor RAP2-11-like was also up-regulated. The results showed that amidosulfuron treatment in rapeseed had obvious eliciting effect on the synthesis of endogenous ethylene. Ethylene is commonly associated with cell death, restricting cell elongation and regulation of cell division (Rogers, 2013). Some previous evidences suggested that valine, leucine and isoleucine may be involved in the regulation of cell division (Rost et al., 1990) and sulfonylurea herbicides disturbed cell division in the meristematic tissues of plants (Saja et al., 2016). We also found that amidosulfuron can restrain the raceme elongation (cell growth) in the first days after treatment and the pollen development was terminated before microspore mitosis (the flower bud length of 3–3.5 mm), as various sulfonylurea herbicides did in different cultivars (Yu et al., 2006, 2009, 2017; Yu and He, 2014; Li et al., 2015). The increase of endogenous ethylene in the treated plants may cause frustration on plant growth and development including cell cycle.

### Transcriptomic response to amidosulfuron exposure

The toxicity of ALS-inhibiting herbicide on plant reproduction is seldom investigated until recent year (Yu et al., 2006, 2009; Li et al., 2015; Qian et al., 2015). There is increasing evidence that plant reproduction is a more sensitive endpoint than classical short-term physiological measurements of herbicide toxicity (Qian et al., 2015). Although, the target of sulfonylurea herbicides is ALS, their biological effects on microbes and plants are quite different. Microspore development rely on the coordination of thousands of genes and metabolism pathways. Thus, exposure to the male gametocide may also affect the expression of hundreds of genes. For example, Zhu et al. (2015) found that the 1,088 DETs in wheat anther transcriptome, treated with a gametocide SQ-1, were mainly involved in ribosomes, photosynthesis, respiration, purine and pyrimidine metabolism, amino acid metabolism, glutathione metabolism, RNA transport, ROS, protein processing, and ubiquitin-mediated proteolysis. It was also found that many effects of ALS inhibiting herbicides related to ribosome biogenesis, secondary metabolism, cell wall modification, and cell growth (Das et al., 2010). What's more, imazethapyr treated *Arabidopsis* showed induction of detoxification genes at early time points but induction of the genes related to amino acid biosynthesis, secondary metabolites, and tRNAs at a later stage (Manabe et al., 2007).

Similarly, we found that expression of some detoxification and defense-related genes, for example, drug metabolism by cytochrome P450, were also induced. The genes in such pathways as aminoacyl-tRNA biosynthesis and porphyrin and chlorophyll metabolism were up-regulated in the amidosulfuron treated plants (Table 2), and this may a reaction to compensate the loss of amino acids and chlorophyll. Other genes of photosynthesis and oxidative phosphorylation, metabolism of several amino acids, CO_2_ fixation, citrate cycle, fatty acid metabolism, glycolysis/gluconeogenesis, and amino sugar and nucleotide sugar metabolism were down-regulated. Similar to the results of our present study, amino acid metabolism, citrate cycle, photosynthesis, and metabolism of starch and sugar were altered in imazethapyr-treated rice (Qian et al., 2011). Moreover, Li et al. (2015) suggested that some transcripts related to carbohydrate metabolism, plastid structure and flavonoid synthesis, were significant affected by MES. They assumed that the constraints of carbohydrate and lipid metabolism along with energy deficiency and perturbed network regulation in the development anthers might be the main reason for male sterility induced by MES treatment (Li et al., 2015). Based on the results of our present study, the abnormal regulation of 13 interesting DETs validated via qRT-PCR (Figure 9) indicated functional abnormalities about cell cycle, cell wall structure, tissue autophagy, and photosynthesis. The down-regulation of *HFM1, SDS*, and *PAIR1* intimated a halt of cell cycle (presented by raceme elongation and possible microspore mitosis). The down-regulation of genes encoding glucanase A6, putative cell wall protein LOC106368794, and UGE1 suggested functional abnormalities about microspore release from tetrad and cell wall formation by protein and polysaccharide. Down-regulation of chloroplastic *PPD2* and up-regulation of *ATG8A* supported the observation of sulfonylurea herbicides destructed chloroplast thylakoid and delivery of the autophagosomes to the vacuole, respectively. ATG8 is a key protein involved in autophagosome formation and can be used as a marker for autophagosomes when fused with a fluorescent protein (Pu et al., 2017). *CSTF77* is one of the multiple factors required for polyadenylation and 3′-end cleavage of pre-mRNAs (Liu et al., 2010) and down-regulation of it might affect the process of gene silence. In addition, *ALA6* was involved in transport of phospholipids (McDowell et al., 2015) and hence down-regulation of it might affect the regulation of pollen plasma membrane lipid asymmetry. The down-regulation of *FLA20*, which encodes an important arabinoglactan protein, suggested that pollen wall structure maybe affected by the amidosulfuron treatment.

However, there were also some DETs in our results different from other studies. This difference could be raised by three reasons: different transcriptomic assay methods (microarray hybridizing vs. RNA-seq), different cultivars and tissue samples, and most importantly, different chemical structures. The genes responses toward two structurally related herbicides (primisulfuron-methyl and sulfometuron-methyl) on gene transcription could be differentiated (Duhoux et al., 2015). The expression patterns of a set of 101 genes in *A. thaliana* and *B. napus* were also differentiated among various herbicides of sulfonylurea family or other ALS-inhibitors (Das et al., 2010).

### Mode of action for gametocide amidosulfuron

In our present paper, the gametocide amidosulfuron showed multi-effects on rapeseed in the first several days after the treatment. These evidences showed that severe destruction of rapeseed photosynthesis system and abnormal regulation of cell cycle related genes, and induction of autophagy in anther cells were associated with the CIMS. Although the relationships among ALS protein function and photosynthesis system and cell cycle are not known, the available data in our studies and previous literatures (Rost et al., 1990; Qian et al., 2011; Cheng et al., 2013; Li et al., 2015; Saja et al., 2016; Sun et al., 2016) suggested that disruption of photosynthesis and inhibition of cell cycle are two major phytotoxic effects of amidosulfuron. Down-regulation of some genes about carbohydrate metabolism and sugar transporter also intimated a possible disruption of photoassimilate translocation. The other hypothesis including BCAAs starvation and substrates accumulation could be excluded, as the previous studies (Giardina et al., 1987; Shaner and Singh, 1993; Höfgen et al., 1995; Royuela et al., 2000; Shim et al., 2003).

**Figure 10:**
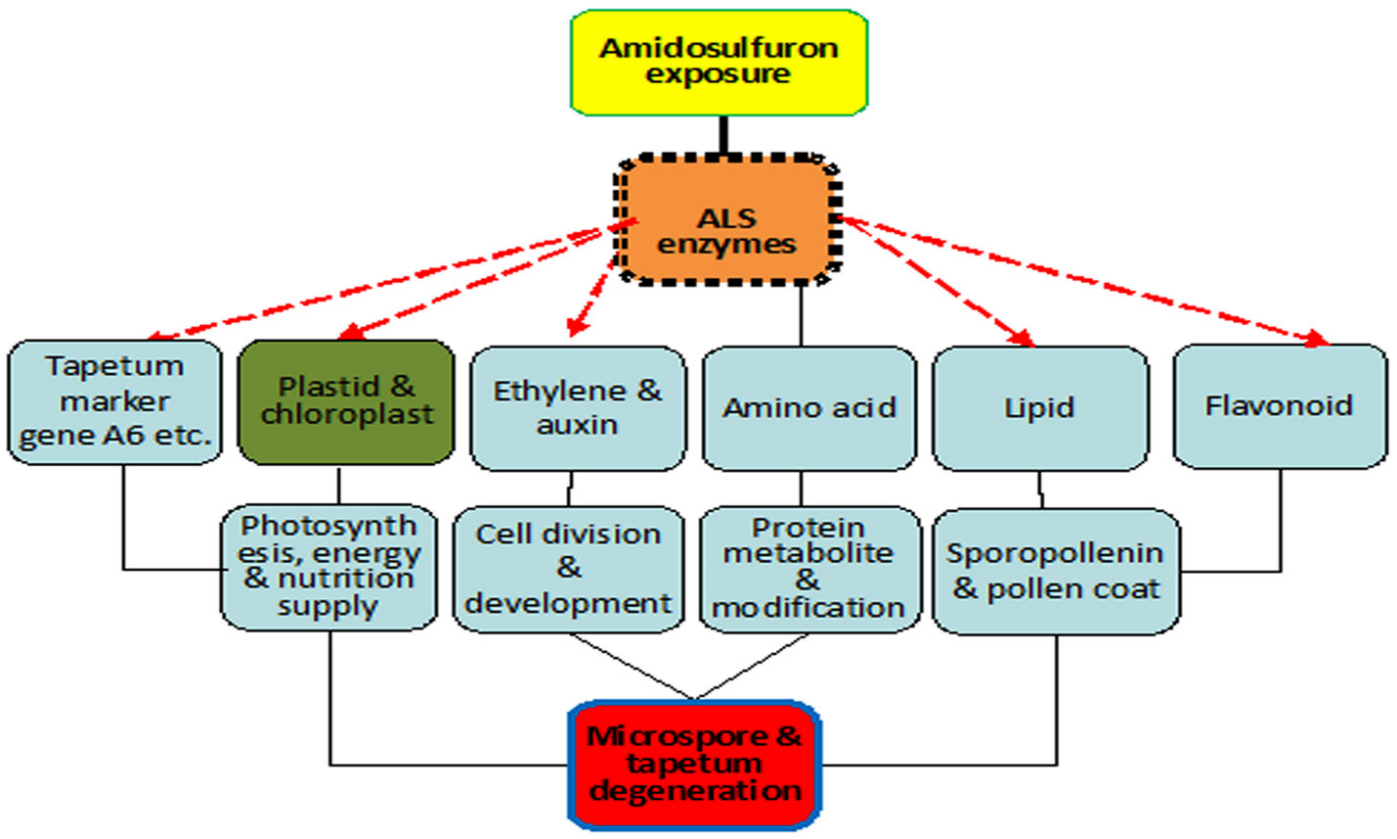
Scheme of mode of action for gametocide amidosulfuron. Inhibition of ALS activity disturbs amino acid metabolism directly and destructs plastid and chloroplast indirectly. Then the treatment affects the expression of some tapetum preferential genes and synthesis of lipid, flavonoid, and pollen coat materials. Consequently, photosynthesis, energy and nutrition supply, cell division and development, and protein metabolism are all influenced by the treatment.

Based on the results in the present paper and previous literatures, a primary model of action for gametocide amidosulfuron can be built (Figure 10) as explained below. Firstly, gametocide amidosulfuron (or other sulfonylurea chemicals) inhibits the activity of the target ALS enzyme. Subsequently, its toxicity will embody multi-level alterations: (1) destruction of plastid and chloroplasts and consequent restriction of the photosynthesis and energy supply, (2) ethylene mediated signal and abnormal cell division and development, (3) imbalance of amino acids, decline of protein biosynthesis, and arrest of DNA duplication and RNA transcription, (4) reduction of lipid and flavonoid synthesis, resulting poor structure of exine, and pollen coat. Especially, these alterations make the tapetum dysfunctional and will not supply the nutrients and components necessary for microspore development. Finally, the microsporogenesis process sensitive to these alterations is suppressed and male sterility happens. However, the detailed linkages among these traits and transcriptional regulation need further studies.

## Conclusion

The results of this study indicated that the male sterility in amidosulfuron treated rapeseed plants may be associated with the defective plastids and impaired photosynthesis at early development stage even if we do not clearly understand the mechanism how an ALS inhibitor destruct plastids and reduce photosynthesis. The aberrant transcriptional regulation of some important genes may disrupt the coordination of development and metabolism and result in dysfunction of tapetal cells and defect of microspores. This combined cytological, transcriptomic, and physiological investigation showed a multi-level mode of action of chemical induced male sterility. This mode for gametocidal effect will also be useful to interpret the herbicidal mechanism of sulfonylurea chemicals.

## Author contributions

Conceived and designed the experiments: CY. Performed the experiments: XL, CY, and JD. Performed the bioinformatics analysis: CY. Contributed materials and analyzed the results: JD, SH, and AX. Wrote the paper: CY. All authors read and approved the final manuscript.

### Conflict of interest statement

The authors declare that the research was conducted in the absence of any commercial or financial relationships that could be construed as a potential conflict of interest.

## Supplementary material

### Availability of supporting data

The dataset supporting the results of this article is available in NCBI's Gene Expression Omnibus under accession GSE69679 and Transcriptome Shotgun Assembly accession GDFQ00000000. Other supporting data are included within the article and its additional files.

Table S1 Primers for *ALS* genes and 13 selected differentially expressed transcripts.

Data Sheet S1 The differentially expressed transcripts (DETs) between amidosulfuron-treatment and the control. All up-regulated and down-regulated DETs in amidosulfuron-treatment were listed. The DETs were selected with FDR <0.05 and fold change ≥2. The gene name and symbol, RPKM value, fold change, *p*-value, gene sequence, GO term, KEGG pathway, BLAST hit in NCBI database, and functional annotation in Uniprot database were included. Fold change is calculated as Log_2_ of ratio (Mean_RPKM_DAT/Mean_RPKM_F).

Data Sheet S2 GO terms corresponding to differentially expressed transcripts.

## Abbreviations

ALS: acetolactate synthase
BCAA: branched-chain amino acids
CIMS: chemically induced male sterility
DAT: days after treatment
DGE: digital gene expression
DETs: differential expression transcripts
MES: monosulfuron ester sodium
PMC: pollen mother cell
TEM: transmission electron microscopy.
