# Supplementary material for: Acetolactate Synthase-Inhibiting Gametocide Amidosulfuron Causes Chloroplast Destruction, Tissue Autophagy, and Elevation of Ethylene Release in Rapeseed
Source: Front Plant Sci. 2017 Sep 21;8:1625. doi: 10.3389/fpls.2017.01625 (PMC5613135; doi:10.3389/fpls.2017.01625)
Supplement: Table S1 — Primers for ALS genes and 13 selected differentially expressed transcripts. [file Table1.DOC]

**Table S1. Primers for *ALS* genes and** the selected differentially expressed transcripts.

| Symbol | Gene | Accession number | Forward sequence | Reverse sequence | Length (bp) |
| --- | --- | --- | --- | --- | --- |
| *ALS1* | Acetolatate synthase 1 | XM_013793508.1 | ACCCGTCAATGTCGCACCTCCTTC | CCCGAAGTGGCTATGCAGATTCCC | 299 |
| *ALS3* | Acetolatate synthase 3 | XM_009153182.2 | GCTATTCAGACAATGCTGGATACACC | CTGGGAACAAACCAAAAGCAGTACA | 256 |
| *ATG8A* | Autophagy-related protein 8a | comp54610_c0_seq1 | TCTTCTTCGGAGTCCAATCG | CTCTGTCAGGGTACTTCTCTC | 115 |
| *SDS* | Cyclin-SDS | comp54250_c0_seq8 | TCCAGGCTCTTGACGCATTC | ACTATCCGATCACACCCAACTC | 165 |
| *A6* | Glucan endo-1,3-beta-glucosidase A6 | comp52020_c1_seq2 | CAATCTTGCTTGCCAATGCTATC | TGCGAACCAAACACAGAAAGTC | 152 |
| *PAIR1* | PAIR1 | comp60070_c0_seq14 | TGGAGATGGCTGAGGTTATAG | GTTGTGGTGCTCTTGGAAG | 150 |
| *CSTF77* | Cleavage stimulation factor subunit 77 | comp60083_c1_seq30 | CTGTTGACCTAGATTCCGAAGAC | GGCGAGGATTGATAACTCAGAAC | 90 |
| *PPD2* | Chloroplastic psbP domain-containing protein 2 | comp62936_c1_seq4 | ATTGATAGCACGAGAGGAGGAATC | TGGATGGTGGGTTTATGTGAGAG | 189 |
| *HFM1* | ATP-dependent DNA helicase HFM1 | comp56411_c1_seq5 | ATCAGCCTTCACAGTCACCTTAG | ACTATCACAACCTCTCGTATCCAC | 178 |
| *UGE1* | Bifunctional UDP-glucose 4-epimerase/UDP-xylose 4-epimerase | comp60986_c1_seq14 | TGGAGGATTGTTCTGCTGAG | GGTAGGATAGTCATGTCCGTAG | 165 |
| *ALA6* | Phospholipid-transporting ATPase 6 | comp47752_c0_seq1 | GAAGCAGACAACTGGCATTAAG | AAGCAACTTCATCAGGAGACTC | 197 |
| *Wall* | Putative cell wall protein LOC106368794 | comp58979_c3_seq2 | AGTCCGCTAACTTGCTCTG | TGGCTTCGTCTCTGCTAAC | 91 |
| *FLA20* | Fasciclin-like arabinoglactan protein FLA20 | comp48895_c1_seq1 | CCATCACGCACGATAGCAATCC | CTCCTTCTCCGACCAGCAGTC | 114 |
| *bHLH91* | Transcription factor bHLH91 | comp62299_c0_seq4 | CCAAGGAAGACCAAGAAGTAGAAG | CATAGTAGCCGCCGTTGTTG | 142 |
| *A9* | Tapetum-specific A9 | comp63150_c1_seq3 | CTGGAGACCGTACCTATG | TTGTTAGTTGCTTGGAGAG | 143 |
| *BnActin7* | Beta-actin 7 | EV220887.1 | CGCGCCTAGCAGCATGAA | GTTGGAAAGTGCTGAGAGATGCA | 101 |
